# The *‘outside to inwards’* approach in the spaghetti wrist

**DOI:** 10.1016/j.amsu.2020.05.047

**Published:** 2020-06-06

**Authors:** Luke Geoghegan, Dariush Nikkhah

**Affiliations:** a Section of Vascular Surgery, Department of Surgery and Cancer, Imperial College London. London, UK; b Department of Plastic and Reconstructive Surgery, Royal Free NHS Foundation Trust. London, UK

## Abstract

• The ‘spaghetti wrist’ is a complex injury pattern.
• Emptying the forearm and carpal tunnel permits repair accurate repair of volar structures working from deep to superficial.
• This pragmatic approach permits better anatomical appreciation and sequence of repair.

## 1 Background

Deep lacerations to the volar aspect of the distal forearm may result in a ‘spaghetti wrist’ pattern of injury with multiple lacerations to flexor tendons, nerves and vasculature. The term ‘spaghetti wrist’ was originally coined by Puckett and Meyer to describe a volar forearm laceration with three or more injured structures [1], and Koshy et al. recently described a classification system based on the extent of injury [2]. Accurate repair is reliant on knowledge of anatomy and a systematic method of repair. This reduces risks of inadvertently repairing wrong structures and prolonged Tourniquet times.

## 2 Technique

**Fig. 1:**
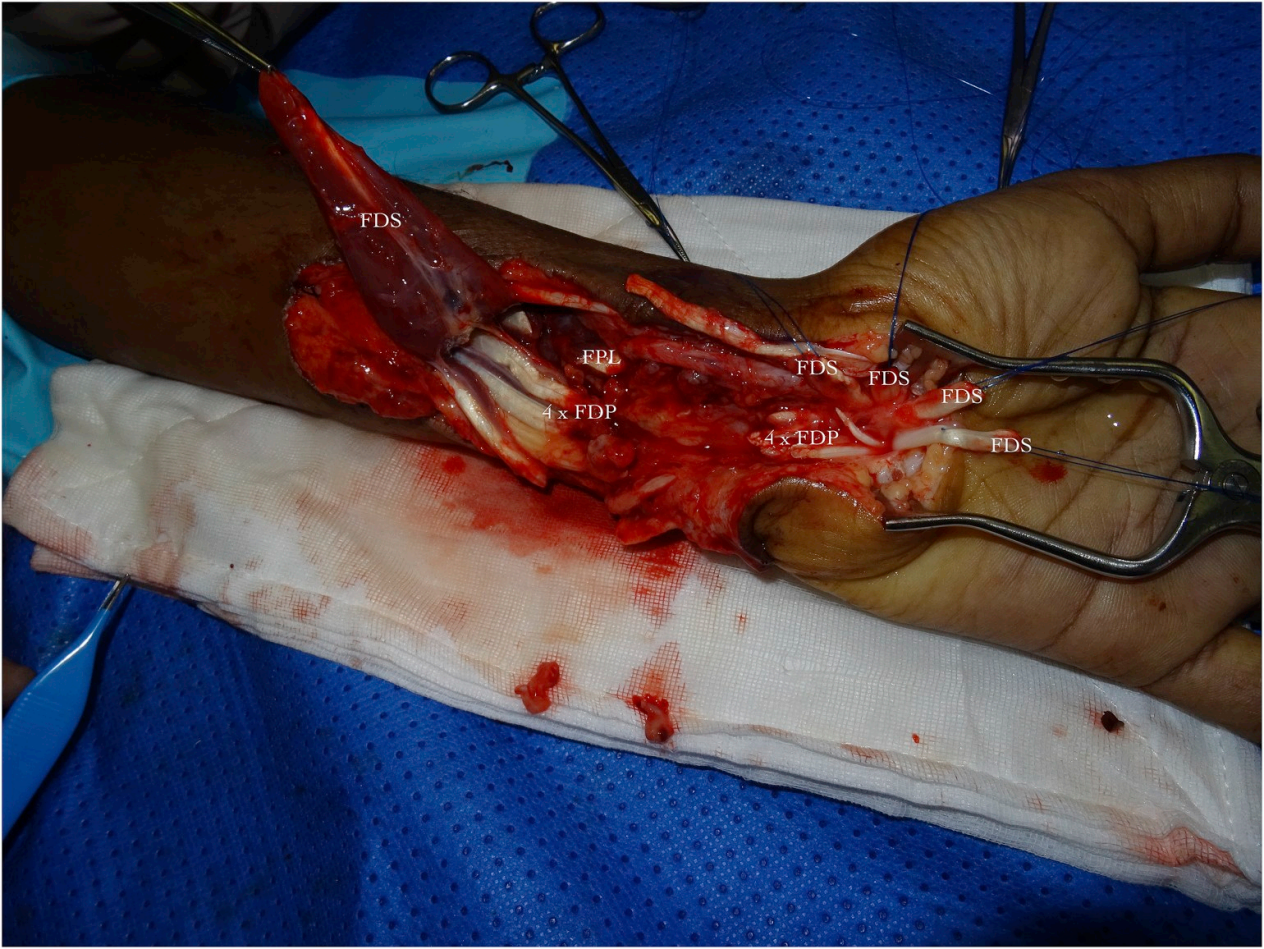
Every structure in the wrist has been divided except for the Flexor Carpi Radialis (FCR) and radial artery. All the structures have been pulled outside of the wrist to align all the key deep structures before repair.

In cases where 10 or more structures are divided (Fig. 1); we use the *‘outside to inwards’* approach, where the forearm or carpal tunnel is emptied of divided structures, placing them *outside* the zone of repair and then working *inwards* for repair. We tag the distal cut ends of the FDS tendons with a half Kessler outside of the zone of repair and proximally with mosquito clips; moving structures outside will therefore leave volar structures for repair. The volar FDP tendons are distinct anatomically as they all lie in the same plane whilst the FDS tendons of the index and little lie volar to the middle and ring FDS tendons.

## 3 Discussion

**Fig. 2:**
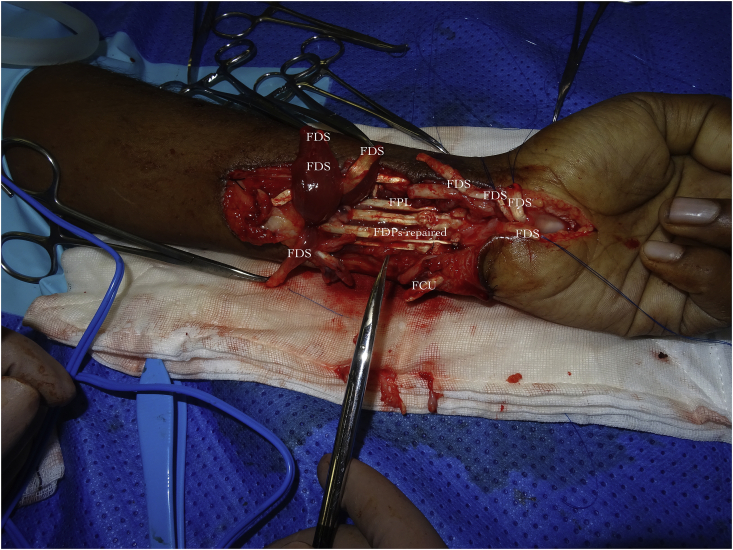
All Flexor Digitorum Profundus (FDP) tendons have all be repaired, one can see all the structures that have been placed outside the zone of repair. This has enabled the surgeon to accurately join up remaining structures which are tagged.

The outside to inwards approach permits better visualisation and anatomical alignment of volar structures. The repairs can then be started from deep to superficial and as demonstrated the anatomy is better appreciated with this sequence of repair (Fig. 2).

## 4 Provenance and peer review

Not commissioned, externally peer reviewed.

## Funding

No funding was received for the conduct of this study.

## Ethical approval

Ethical approval was not required for this study.

## Consent

N/A.

## Author contribution

LG and DN were both involved in the study concept and writing the paper.

## Registration of research studies

This study was not registered with the research registry as it did not involve human subjects.

## Guarantor

I, Luke Geoghegan, accept full responsibility for the work.

## Declaration of competing interest

The authors declared no potential conflicts of interest with respect to the research, authorship, and/or publication of this article.

## References

[1] Puckett C.L., Meyer V.H. (1985). Results of treatment of extensive volar wrist lacerations: the spaghetti wrist. Plast. Reconstr. Surg..

[2] Koshy K., Prakash R., Luckiewicz A., Alamouti R., Nikkhah D. (2018). An extensive volar forearm laceration – the spaghetti wrist: a systematic review. JPRAS Open.

